# Functional and structural reverse myocardial remodeling following transcatheter aortic valve replacement: a prospective cardiovascular magnetic resonance study

**DOI:** 10.1186/s12968-022-00874-0

**Published:** 2022-07-28

**Authors:** Torben Lange, Sören J. Backhaus, Bo Eric Beuthner, Rodi Topci, Karl-Rudolf Rigorth, Johannes T. Kowallick, Ruben Evertz, Moritz Schnelle, Susana Ravassa, Javier Díez, Karl Toischer, Tim Seidler, Miriam Puls, Gerd Hasenfuß, Andreas Schuster

**Affiliations:** 1 Department of Cardiology and Pneumology, University Medical Center Göttingen, Georg-August University, Robert-Koch-Straße 40, 37075 Göttingen, Germany; 2 German Center for Cardiovascular Research (DZHK), Partner Site Göttingen, Göttingen, Germany; 3 Department of Diagnostic and Interventional Radiology, University Medical Center Göttingen, Georg-August University, Göttingen, Germany; 4 Department of Clinical Chemistry, University Medical Center Göttingen, Georg-August University, Göttingen, Germany; 5 Program of Cardiovascular Diseases, Centre for Applied Medical Research, University of Navarra, Pamplona, Spain; 6 IdiSNA, Navarra Institute for Health Research, Pamplona, Spain; 7 Cluster of Excellence “Multiscale Bioimaging: From Molecular Machines to Networks of Excitable Cells” (MBExC), University of Göttingen, Göttingen, Germany

**Keywords:** Cardiac magnetic resonance imaging, Transcatheter aortic valve replacement, Myocardial remodeling, Assessment of myocardial function and structure

## Abstract

**Background:**

Since cardiovascular magnetic resonance (CMR) imaging allows comprehensive quantification of both myocardial function and structure we aimed to assess myocardial remodeling processes in patients with severe aortic stenosis (AS) undergoing transcatheter aortic valve replacement (TAVR).

**Methods:**

CMR imaging was performed in 40 patients with severe AS before and 1 year after TAVR. Image analyses comprised assessments of myocardial volumes, CMR-feature-tracking based atrial and ventricular strain, myocardial T1 mapping, extracellular volume fraction-based calculation of left ventricular (LV) cellular and matrix volumes, as well as ischemic and non-ischemic late gadolinium enhancement analyses. Moreover, biomarkers including NT-proBNP as well as functional and clinical status were documented.

**Results:**

Myocardial function improved 1 year after TAVR: LV ejection fraction (57.9 ± 16.9% to 65.4 ± 14.5%, p = 0.002); LV global longitudinal (− 21.4 ± 8.0% to -25.0 ± 6.4%, p < 0.001) and circumferential strain (− 36.9 ± 14.3% to − 42.6 ± 11.8%, p = 0.001); left atrial reservoir (13.3 ± 6.3% to 17.8 ± 6.7%, p = 0.001), conduit (5.5 ± 3.2% to 8.4 ± 4.6%, p = 0.001) and boosterpump strain (8.2 ± 4.6% to 9.9 ± 4.2%, p = 0.027). This was paralleled by regression of total myocardial volume (90.3 ± 21.0 ml/m^2^ to 73.5 ± 17.0 ml/m^2^, p < 0.001) including cellular (55.2 ± 13.2 ml/m^2^ to 45.3 ± 11.1 ml/m^2^, p < 0.001) and matrix volumes (20.7 ± 6.1 ml/m^2^ to 18.8 ± 5.3 ml/m^2^, p = 0.036). These changes were paralleled by recovery from heart failure (decrease of NYHA class: p < 0.001; declining NT-proBNP levels: 2456 ± 3002 ng/L to 988 ± 1222 ng/L, p = 0.001).

**Conclusion:**

CMR imaging enables comprehensive detection of myocardial remodeling in patients undergoing TAVR. Regression of LV matrix volume as a surrogate for reversible diffuse myocardial fibrosis is accompanied by increase of myocardial function and recovery from heart failure. Further data are required to define the value of these parameters as therapeutic targets for optimized management of TAVR patients.

*Trial registration* DRKS, DRKS00024479. Registered 10 December 2021—Retrospectively registered, https://www.drks.de/drks_web/navigate.do?navigationId=trial.HTML&TRIAL_ID=DRKS00024479

## Introduction

Aortic stenosis (AS) is the most common cardiac valve disease [1]. With an estimated prevalence of more than 7.5 million among those over 75 years in North America and Europe, valve replacement and especially transcatheter aortic valve replacement (TAVR) plays a key role in patient management with rapidly increasing numbers of procedures [2, 3]. Over the past years indications and strategies for TAVR have evolved. Initially, TAVR was nearly exclusively considered for patients at high-risk [4–6], but several trials have proven utility and no inferiority regarding outcome compared to surgery even in inter-mediate and low-risk patients [7–9]. With a constantly-widening collective of AS patients undergoing TAVR, adapted periprocedural monitoring and in particular approaches for the evaluation and observation of myocardial remodeling processes are increasingly required for a successful patient management. 

In this context, especially cardiovascular magnetic resonance (CMR) imaging is of growing importance as an appropriate non-invasive tool for comprehensive myocardial analyses including strain and tissue analyses. Previous CMR studies have demonstrated detection of volumetric functional improvement following TAVR and recently applied CMR imaging to identify dynamic structural changes of myocardial compartments assessing reverse myocardial remodeling and recovery from AS [10, 11]. Importantly, CMR imaging allows non-invasive assessment of myocardial fibrosis, which is of growing relevance as another parameter for ventricular failure in AS patients and possesses important prognostic informations [12, 13]. Recently, a novel laboratory parameter measuring irreversible myocardial fibrosis by the ratio of serum carboxy-terminal telopeptide of collagen type I to serum matrix metalloproteinase-1 (CITP:MMP1) has been introduced [14].

However, combined data of comprehensive CMR-derived functional and structural reverse myocardial remodeling, their interplay and especially their associations with various biomarkers including indicators of reversible or cross-linked collagen deposition in AS patients following TAVR are scarce.

Therefore, the aim of this study was to non-invasively quantify and characterize myocardial tissue composition as well as cardiac function before and 1 year after TAVR and to contextualize analyzed imaging parameters with clinical and laboratory biomarkers for a more comprehensive and in-depth understanding of reverse myocardial remodeling processes in patients with severe symptomatic AS undergoing TAVR.

## Methods

### Study population

**Fig. 1:**
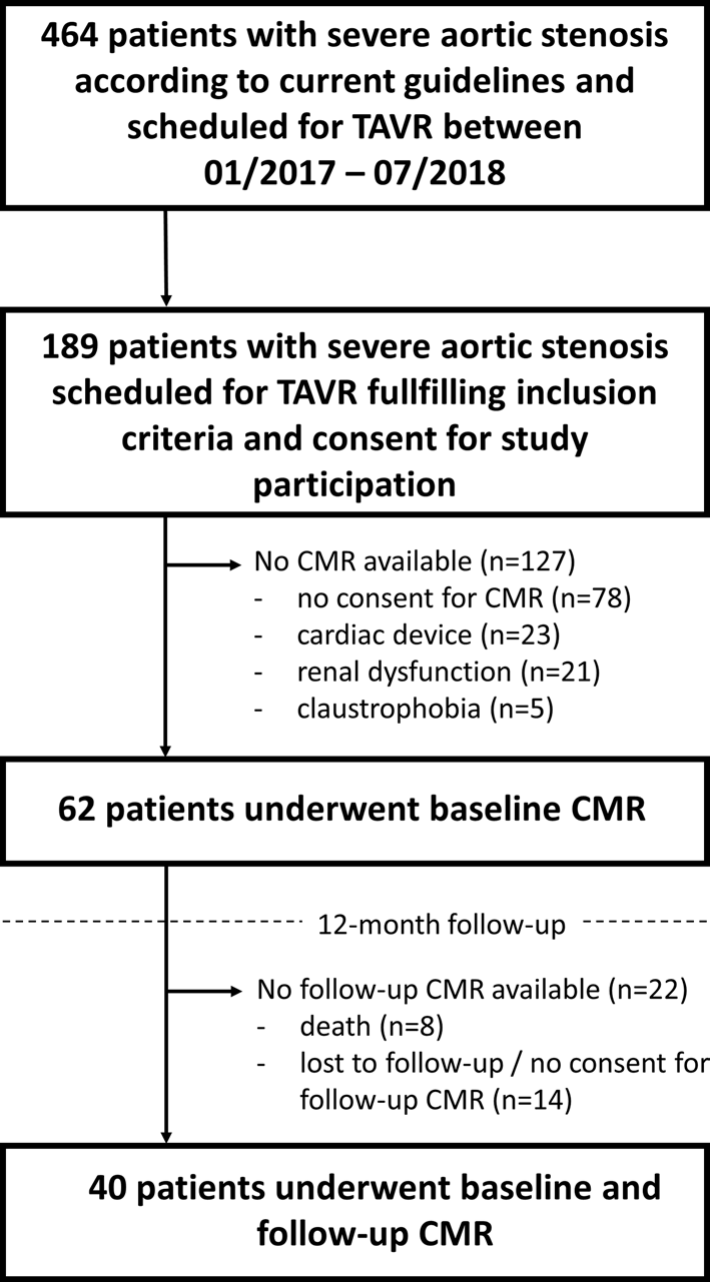
Study Flow-chart. Study flow-chart of patients scheduled for transcatheter aortic valve replacement (TAVR) at the University Hospital Goettingen between 01/2017 and 07/2018 according to current guideline recommendations [15]. Patients who met inclusion criteria [16] were invited to participate the study including cardiovascular magnetic resonance (CMR) imaging if practicable

The study population consisted of 40 patients who underwent TAVR between January 2017 and July 2018 at the Heart Center of the University Medical Center Göttingen, Germany (Fig. 1). All patients had severe symptomatic AS and were scheduled for transfemoral TAVR after consensus decision by an interdisciplinary heart team [15]. CMR imaging was performed on average 1 (± 1) day before TAVR. To quantify functional capacity and quality of life, a 6-minute walk test (6MWT) was performed, N-terminal pro-hormone brain natriuretic peptide (NT-proBNP) levels were determined and Minnesota Living with Heart Failure Quality of Life® (MLHFQ) score as well as New York Heart Association (NYHA) class were captured [16]. Clinical follow-up including CMR imaging, 6MWT, MLHFQ, NYHA class and laboratory testing was performed 379 (± 38) days after TAVR.

Furthermore, at baseline the ratio CITP:MMP1 as an inverse index of myocardial collagen cross-linking reflecting irreversible myocardial fibrosis was analyzed with low CITP:MMP1 ratios suggesting dominance of irreversible collagen deposition and high ratios suggesting potentially reversible collagen formation [14]. Analyses were performed by an ELISA based assay for CITP (Orion Diagnostica, Espoo, Finland), and an alphaLISA for quantification of total serum MMP-1 levels (PerkinElmer, Waltham, Massachusetts, USA) [17].

All patients gave written informed consent before participation. The study was approved by the local ethics committee and conducted according to the principles of the Helsinki Declaration. 

### CMR Imaging protocol

CMR examinations of enrolled patients were performed on a 3T CMR system (Magnetom Skyra, Siemens Healthineers, Erlangen, Germany) using a 32-channel cardiac surface receiver coil. The protocol included electrocardiography-gated balanced steady state free precession (bSSFP) images of long-axis two, three- and four-chamber views as well as short-axis (SAx) stacks. Typical bSSFP image parameters were as follows: 25 frames per cardiac cycle, time of echo (TE) 1.5 ms, time of repetition (TR) 55 ms, flip angle 55°, 7 mm slice thickness with 7.7 mm inter-slice gap. For T1-mapping, conventional 5(3)3 Modified Look-Locker Inversion Recovery (MOLLI) sequences (FOV of 360 × 306.6mm^2^, in-plane resolution 1.41 × 1.41 × 8mm^3^, TR 280 ms, TE 1.12 ms, TI 180 ms, flip angle 35°, bandwidth 1085 Hz/pixel with total acquisition of 11 heart beats) were performed before and 20 min after admission of a gadolinium contrast bolus (0.15 mmol/kg bodyweight). Moreover, phase-sensitive inversion-recovery-gradient echo sequences were acquired 15–20 min after the gadolinium bolus injection for late gadolinium enhancement (LGE) assessment (TR 700 ms; TE 1.24 ms; flip angle 40°; slice thickness 7 mm, with individually adjusted inversion times typically between 300 and 400 ms).

### CMR image analysis

Dedicated post-processing software was used for volumetric assessment including left ventricular (LV) mass, LV end-diastolic volume (LVEDV) and LV end-systolic volumes (LVESV) as well as LV ejection fraction (LVEF) (QMass, Version 8.1.48.2; Medis Medical Imaging, Leiden, the Netherlands). For ventricular volumetric analyses, epi- and endocardial borders were manually delineated in SAx stacks covering the LV from base to apex. To ensure better comparability, total LV myocardial volume (tLV myocardial volume) was calculated dividing the automatically calculated LV mass by the specific gravity of myocardium (1.05 g/ml) and consequently all results of myocardial tissue analyses are reported in indexed volumes (ml/m^2^). 

**Fig. 2:**
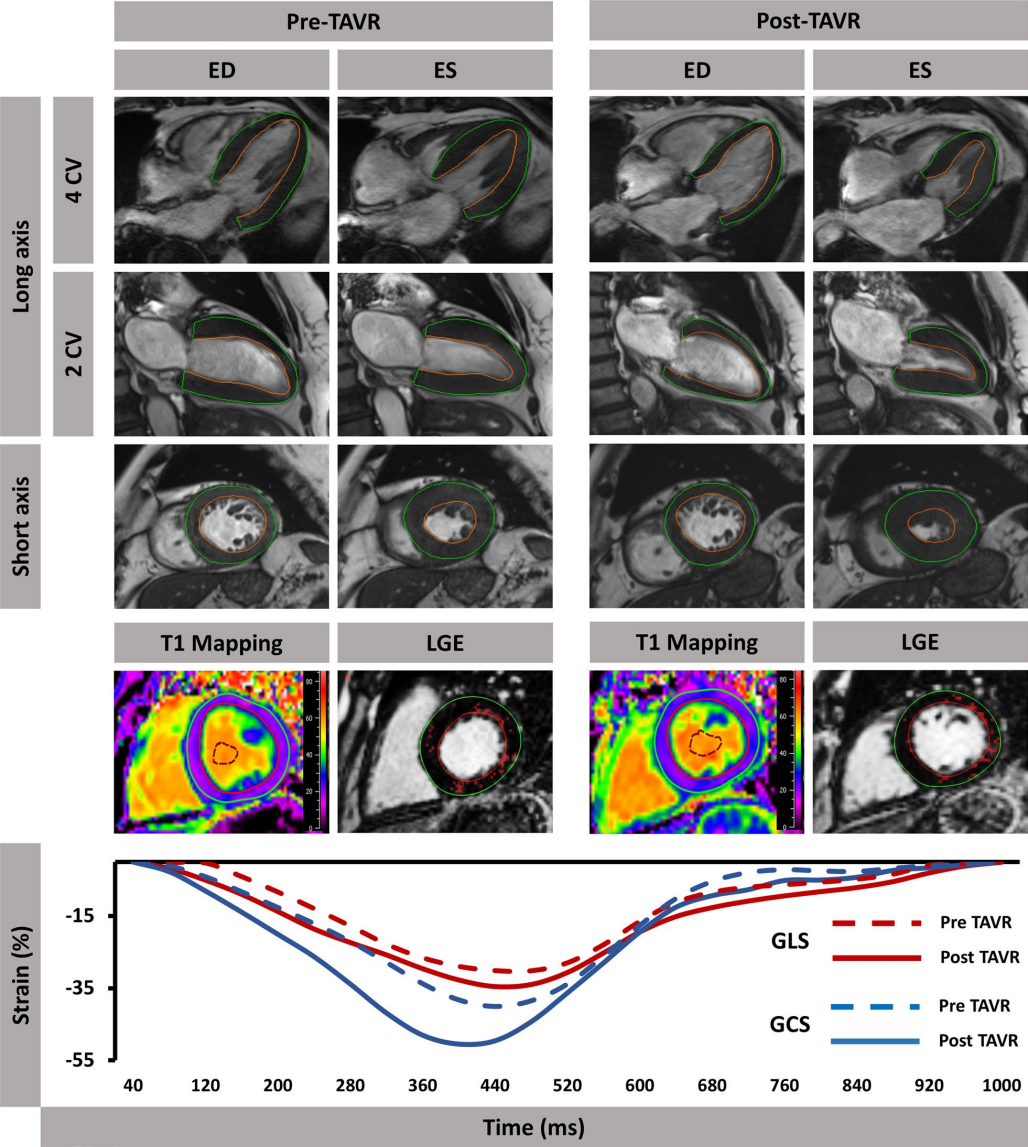
Feature-Tracking in TAVR patients. Feature-Tracking of AS patients before and after TAVR. Myocardial contours were traced in end-diastole (ED) as well as end-systole (ES) of long axis 2- and 4-chamber views (CV) and short axis orientations. T1 mapping and scar analyses using late-gadolinium-enhancement (LGE) sequences were performed in short axis images as well. Graphs of global ventricular strain parameters (longitudinal [GLS] and circumferential [GCS]) over a whole cardiac cycle are depicted. Dotted lines indicate strain performance before intervention, solid lines represent strain characteristics 1 year after TAVR

CMR-feature tracking (CMR-FT)-derived ventricular strain analyses were performed in bSSFP images using dedicated evaluation software that has been validated in numerous previous studies (QStrain, Version 2.0.48.2; Medis Medical Imaging,) [18, 19]. LV epi- and endocardial borders were manually traced at end-diastole and -systole with papillary muscles being included in LV myocardial mass whereas endocardial trabecular tissue was excluded. Global longitudinal strain (GLS) was calculated from 2-, 3- and 4-chamber long axis orientations. Furthermore, left atrial (LA) endocardial contours were delineated in 2- and 4-CV images to quantify LA reservoir (LA Es), conduit (LA Ee) and boosterpump strain (LA Ea). SAx slices were analyzed on basal, midventricular and apical levels to acquire global circumferential strain (GCS) (Fig. 2) as previously described [20, 21].

**Fig. 3:**
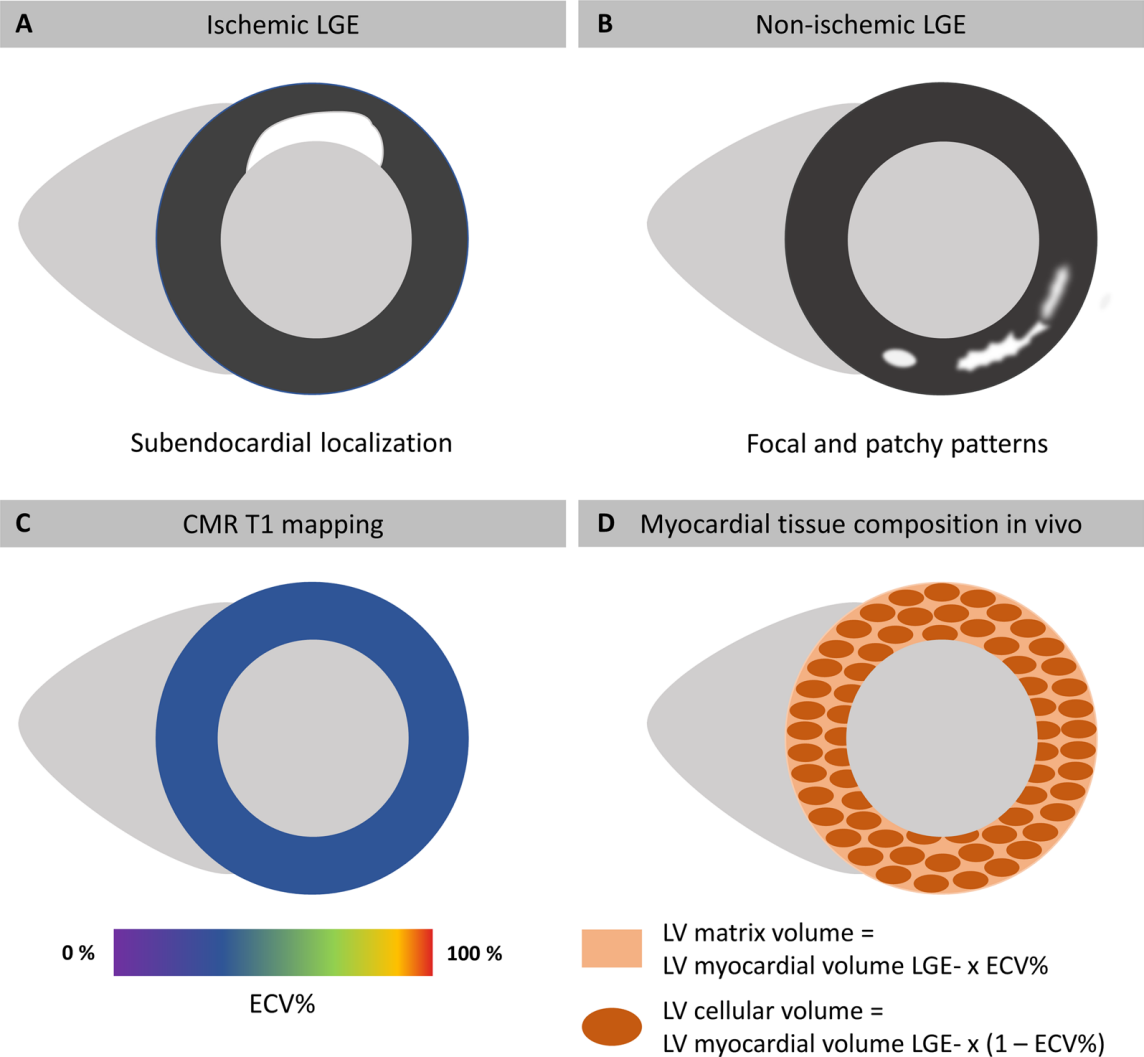
CMR-based myocardial tissue characterization. **A** CMR-derived ischemic late-gadolinium-enhancement (LGE) generally extending from the subendocardium representing ischemia caused irreversible myocardial damage. **B** Focal and patchy LGE pattern across the myocardium illustrating non-ischemic LGE. **C** Based on CMR-derived T1 mapping technique extracellular volume fraction (ECV) as the relative extracellular share of myocardial volume can be calculated. **D** Both left ventricular (LV) matrix and cellular volume portions of the in vivo myocardium can be calculated from the products of LV myocardial volume without LGE (LV myocardial volume LGE-) and ECV for LV matrix volume or [1 – ECV] for LV cellular volume

T1-mapping for the assessment of extracellular volume fraction (ECV) reflecting the volume fraction of the myocardial tissue that is not occupied by cells was performed based on motion-corrected MOLLI sequences using QMap software (Version 2.2.36; Medis Medical Imaging,). First, correct overlapping of T1 weighted images was ensured by the image analyst before drawing a region of interest excluding any LGE areas. Afterwards, the delineated region of interest was propagated to all T1 weighted images and a subsequent careful revision of the delineation on all underlying T1 weighted images was performed to avoid partial volume effects caused by the blood pool or adjacent non-myocardial structures. According to guideline recommendations ECV was defined as follows: ECV = (1 – hematocrit) * [Δ R1 myocardium]/[Δ R1 blood] [22]. LGE analyses were performed in SAx images of inversion recovery sequences contouring epi- and endocardial borders and defining a 3-standard deviation (SD) threshold of signal intensity for the detection of enhancement in % of tLV myocardial volume [23]. LGE patterns were further divided into ischemic LGE with enhanced areas generally extending from the subendocardium with varying transmurality and non-ischemic LGE patterns comprising focal as well as patchy enhancements within the myocardium (Fig. 3A and B). Both ischemic LGE and non-ischemic LGE areas were excluded from T1-mapping and final ECV evaluations. Furthermore, LV myocardial volume without LGE (LV myocardial volume LGE-) was calculated by subtracting ischemic LGE and non-ischemic LGE volumes from tLV myocardial volume. Subsequently, LV cellular and matrix volumes were calculated from the product of LV myocardial volume LGE- and ECV for LV matrix volume and (1 – ECV) for LV cellular volume (Fig. 3C and D). All analyses were performed by an observer blinded to clinical and imaging characteristics respectively.

### Statistical analysis

Categorical variables are reported as absolute numbers and percentages. Continuous variables are presented as mean ± standard deviation if normally distributed and as median with interquartile range (IQR) if non-normally distributed. The Shapiro–Wilk test was used to test for normal distribution. Data correlations are reported using Pearson’s correlation coefficient for normal distributed values or spearman´s rank correlation coefficient technique for skewed data. Dependent parameters of pre- and post-TAVR examinations were compared using Wilcoxon signed rank test. Delta values (Δ) of CMR parameters before and after TAVR were calculated by subtracting follow-up values from baseline values and are reported in absolute numbers. Independent variables were compared by applying the Mann–Whitney-U test. Linear regression analyses were performed to model the relationship between diffuse myocardial fibrosis and alterations of strain parameters and to identify predictors of functional and structural changes. For right skewed data log-transformation was performed to reach normal distribution before regression analyses. The CITP:MMP1 ratio was corrected for estimated glomerular filtration rate in statistical analyses. All p-values are provided two sided and considered as statistically significant with an alpha level of 0.05 and below. For all statistical calculations SPSS (v 25 for Windows 10; Statistical Package for the Social Sciences, International Business Machines, Inc., Armonk, New York, USA) was used.

## Results

### Study population

The study population predominantly consisted of male patients (60%) with an average age of 78.5 ± 6.6 years, while women were slightly older (80.5 ± 5.0 years). All patients had echocardiographically proven severe AS with a mean AV area/body surface area of 0.4 cm^2^/m^2^ (0.3–0.5). Amongst the study cohort there were a few missing values in the data set due to insufficient CMR image quality (39/40 strain and volumetric analyses, 36/40 ECV analyses, 38/40 LGE analyses), missing laboratory samples (39/40 CITP:MMP1) or inability to walk (37/40). These data were assumed missing at random and complete case analyses were conducted. Cardiovascular risk factors such as hypertension (87.5%) or hyperlipoproteinemia (57.5%) were present in the majority of patients. More detailed baseline characteristics are displayed in Table 1. During the follow-up period 9 patients had a rehospitalization within a mean time of 112 ± 68 days after TAVR intervention due to acute myocardial infarction (n = 1), elective percutaneous coronary revascularization (n = 2), symptomatic tachyarrhythmia (n = 1), heart failure (n = 1), hypertensive crisis (n = 1), secondary bleeding (n = 1) and thromboembolic complications (n = 2). Measuring functional capacity and quality of life after TAVR, MLHFQ decreased notably (p < 0.001), distance in 6MWT improved (p = 0.001) and NYHA class of TAVR patients ameliorated (-0.6 points in NYHA class, p < 0.001). NT-proBNP decreased 1 year after TAVR compared to baseline (2456 ± 3002 ng/L at baseline vs. 988 ± 1222 ng/L at follow-up, p = 0.001) (Table 2).

**Table 1:** Baseline characteristics

Parameter	Total (n = 40)
Sex (f/m)	(16/24)
Age (years)	78.6 ± 6.0
BMI (kg/m^2^)	28.6 (24.5–31.1)
Pmean (mmHg)	38.0 (31.5–51.3)
Vmax (m/s)	4.1 (3.6–4.4)
AVA/BSA (cm^2^/m^2^)	0.4 (0.3–0.5)
Euroscore II (%)	3.6 (2.2–6.9)
Cardiovascular Risk Factors	
Hypertension (n)	35 (87.5%)
Diabetes (n)	16 (40.0%)
Dyslipidemia (n)	23 (57.5%)
Current smoking (n)	1 (2.5%)
Atrial fibrillation (n)	7 (17.5%)
Coronary artery disease (n)	23 (57.5%)

Baseline characteristics of the study cohort. Two patients did not fit within a single AS subgroup and consequently were not listed amongst the subgroups but included in the remaining baseline characteristics. AVA: aortic valve area; BMI: body mass index; BSA: body surface area

**Table 2:** Changes 1 year after TAVR

Parameter	Pre-TAVR	1 year Post-TAVR	p-value
NYHA class, n (%)			** < 0.001**
I	4 (10.0)	10 (25.0)	
II	10 (25.0)	23 (57.5)	
III	26 (65.0)	6 (15.0)	
IV	/	1 (2.5)	
NT-proBNP, ng/L	1452 (590–3249)	568 (239–1002)	**0.001**
CITP:MMP1 ratio	2.1 (1.7)	/	
6-minute walk test, meters	249.6 (95.9)	317.0 (98.6)	**0.001**
MLHFQ, points	29.9 (16.1)	20.1 (18.2)	** < 0.001**
Functional parameters			
LVEF, %	58.7 (45.2–71.4)	67.1 (56.3–77.5)	**0.002**
LVEDVI, ml/m^2^	82.9 (65.5–101.8)	71.3 (64.9–84.7)	**0.018**
LVESVI, ml/m^2^	33.8 (18.1–52.2)	22.2 (14.8–32.5)	**0.002**
LV mass index, g/m^2^	94.9 (22.1)	77.1 (17.9)	**< 0.001**
GLS, %	− 21.4 (8.0)	− 25.0 (6.4)	**< 0.001**
GCS, %	− 36.9 (14.3)	− 42.6 (11.8)	**0.001**
LA Es, %	13.3 (6.3)	17.8 (6.7)	**0.001**
LA Ee, %	5.5 (3.2)	8.4 (4.6)	**0.001**
LA Ea, %	8.2 (4.6)	9.9 (4.2)	**0.027**
Tissue characterization			
T1 myocardium native, ms	1310.6 (60.2)	1288.4 (55.6)	0.11
ECV, %	27.1 (2.9)	29.3 (3.3)	**0.005**
tLV myocardial volume indexed, ml/m^2^	90.3 (21.0)	73.5 (17.0)	**< 0.001**
Non-invasive LGE, % tLV myocardial volume	12.0 (6.1)	10.6 (7.1)	0.18
Non-invasive LGE volume indexed, ml/m^2^	7.6 (5.6–13.4)	7.1 (4.9–8.9)	**0.007**
Ischemic LGE, % tLV myocardial volume	2.5 (6.5)	2.9 (6.6)	0.17
Ischemic LGE volume indexed, ml/m^2^	2.4 (6.0)	2.7 (6.0)	0.17
Total LGE, ml/m^2^	9.2 (5.6–14.0)	7.7 (6.0–13.1)	**0.018**
LV myocardial volume LGE- indexed, ml/m^2^	75.8 (18.5)	63.3 (16.4)	**< 0.001**
LV Cellular volume indexed, ml/m^2^	55.2 (13.2)	45.3 (11.1)	**< 0.001**
LV Matrix volume indexed, ml/m^2^	20.7 (6.1)	18.8 (5.3)	**0.036**

Changes of dependent parameters pre- and post-TAVR (± standard deviation or interquartile range) were compared using Wilcoxon signed rank test. P-values in bold indicate statistical significance. CITP:MMP1 ratio: ratio of serum carboxy-terminal telopeptide of collagen type I to serum matrix metalloproteinase-1; ECV: extracellular volume fraction; GCS: global circumferential strain; GLS: global longitudinal strain; LA Es: left atrial reservoir strain; LA Ee: left atrial conduit strain; LA Ea: left atrial boosterpump strain; LV: left ventricular; LGE: late-gadolinium-enhancement; LVEDVI: left ventricular end-diastolic volume index; LVESVI: left ventricular end-systolic volume index; LV myocardial volume LGE-: left ventricular myocardial volume without LGE; tLV myocardial volume: total left ventricular myocardial volume; MLHFQ: Minnesota Living with Heart Failure Quality of Life® score; NYHA: New York Heart Association; 3SD: 3-standard deviations

### CMR image analysis of functional parameters

**Fig. 4:**
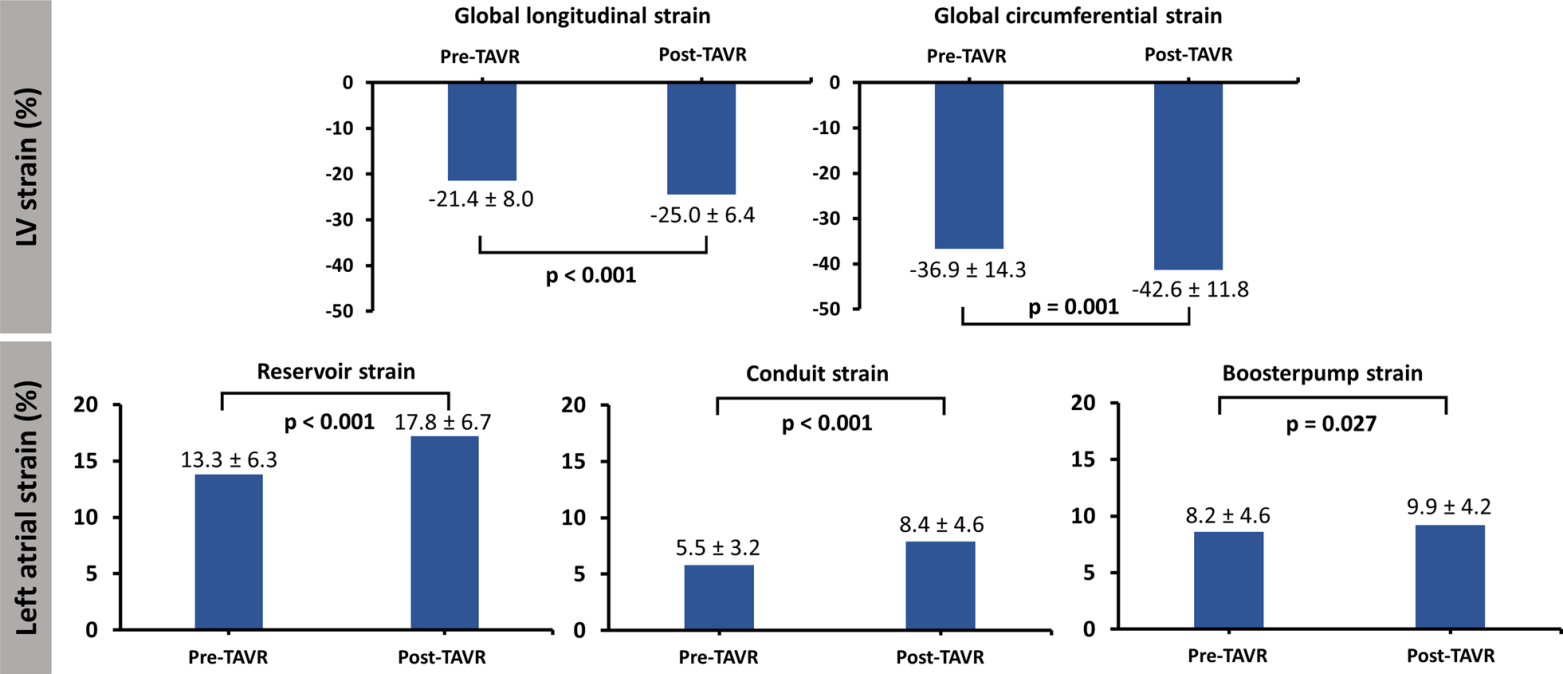
Functional changes 1 year after TAVR. Bar charts of LV global longitudinal (GLS), circumferential radial strain (GCS) as well as atrial reservoir, conduit and boosterpump strain before and 1 year after TAVR. P-values displayed in bold indicate statistical significance

LVEF relatively increased by 13% (from 58.7 [45.2–71.4]% to 67.1 [56.3–77.5]%, p = 0.002) and LV volumes decreased (from 82.9 [65.5–101.8] ml/m^2^ to 71.3 [64.9–84.7] ml/m^2^, p = 0.018 for LVEDV index (LVEDVI), from 33.8 [18.1–52.2] ml/m^2^ to 22.2 [14.8–32.5] ml/m^2^, p = 0.002 for LVESV index (LVESVI) (Table 2). All global LV strain values significantly improved (from − 21.4 ± 8.0% to − 25.0 ± 6.4%, p < 0.001 for GLS and from − 36.9 ± 14.3% to − 42.6 ± 11.8%, p = 0.001 for GCS). Likewise, LA reservoir (from 13.3 ± 6.3% to 17.8 ± 6.7 %, p = 0.001), conduit strain (from 5.5 ± 3.2% to 8.4 ± 4.6%, p = 0.001) and boosterpump function (from 8.2 ± 4.6% to 9.9 =/-4.2%, p = 0.027) increased (Fig. 4).

### CMR image analysis of structural parameters

**Fig. 5:**
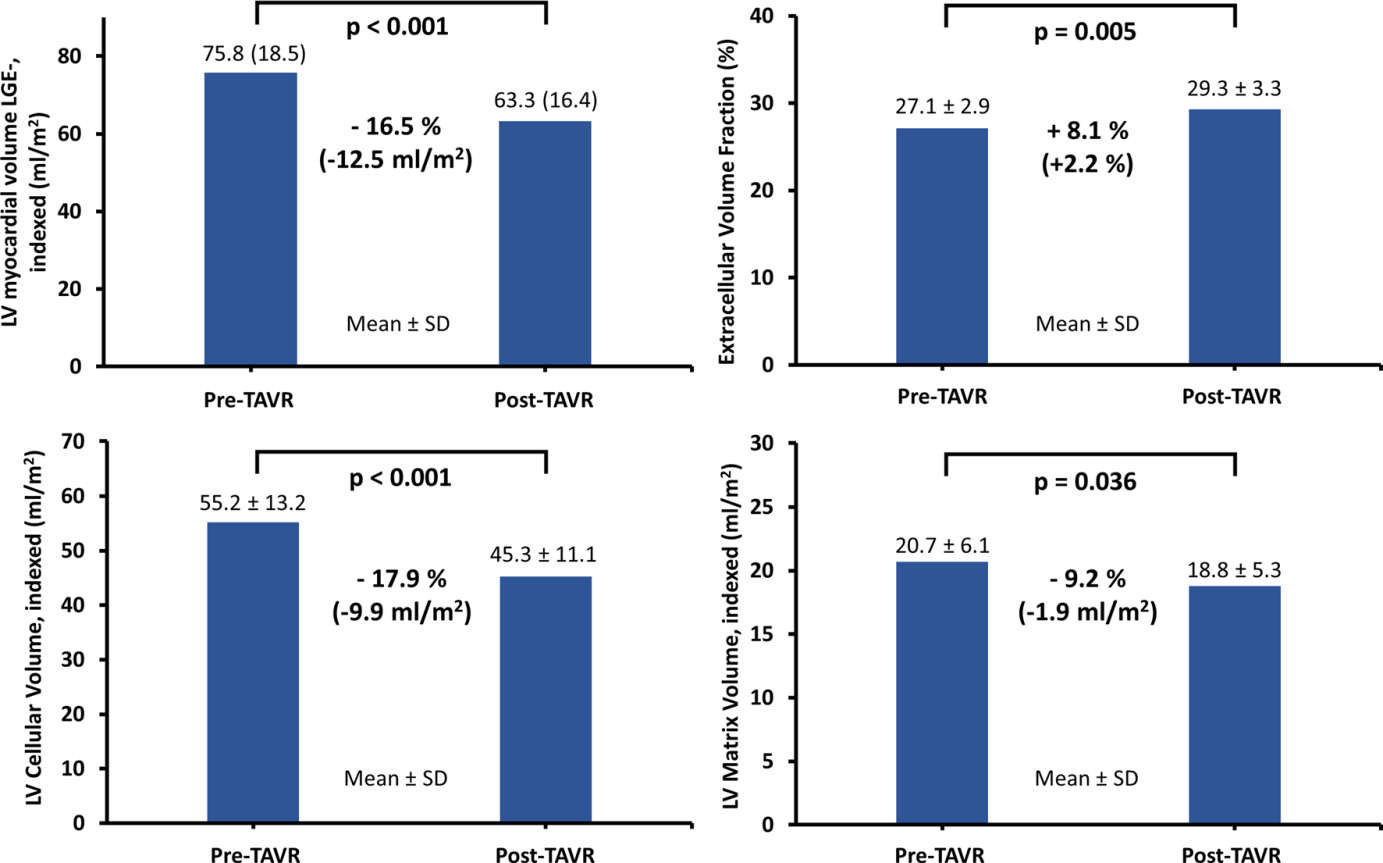
Tissue remodeling 1 year after TAVR. Bar charts of CMR-derived myocardial structure and remodeling 1 year after TAVR. Regression of LV myocardial volume without LGE (LV myocardial volume LGE−) was accompanied by decreases of LV cellular and LV matrix volumes whereas extracellular volume fraction increased. P-values displayed in bold indicate statistical significance

At 1 year follow-up ECV% had considerably increased by 8.1% (from 27.1 ± 2.9% to 29.3 ± 3.3%, p = 0.005) with a relative reduction of tLV myocardial volume by − 18.6% (from 90.3 ± 21.0 ml/m^2^ to 73.5 ± 17.0 ml/m^2^, p < 0.001), LV myocardial volume LGE− by − 16.5% (from 75.8 ± 18.5 ml/m^2^ to 63.3 ± 16.4 ml/m^2^, p < 0.001) and a relatively larger regression of LV cellular volume by − 17.9% (from 55.2 ± 13.2 ml/m^2^ to 45.3 ± 11.1 ml/m^2^, p < 0.001) compared to the regression of LV matrix volume by − 9.2% (from 20.7 ± 6.1 ml/m^2^ to 18.8 ± 5.3 ml/m^2^, p = 0.036) (Table 2, Fig. 5). No significant changes were documented for native myocardial T1 mapping (p = 0.11). In six patients an infarct LGE area was identified, that showed no significant alteration 1 year after TAVR neither in percentage nor absolute volume (p = 0.17, respectively) (Table 2). While there was no statistically significant change of non-infarct LGE in % of tLV myocardial volume (from 12.0 ± 6.1% to 10.6 ± 7.1%, p = 0.18), non-infarct LGE in absolute volume decreased 1 year after TAVR (from 7.6 [5.6–14.0] ml/m^2^ to 7.1 [4.9–8.9] ml/m^2^, p = 0.007).

### Interplay of CMR function and morphology and cardiac biomarkers

Significant associations of all functional LV global strain values with both LV myocardial volume, LV cellular as well as LV matrix volumes were documented (Table 3). In contrast, there were no significant associations of deformation parameters with non-ischemic LGE quantification.

**Table 3:** Correlation of myocardial function and tissue characteristics

Parameter		LV myocardial volume LGE−	LV matrix volume	LV cellular volume	ECV%	Non-ischemic LGE volume
LVEF	r-value	− 0.46	− 0.42	− 0.42	− 0.08	− 0.08
	p-value	**0.016**	**0.035**	**0.032**	0.66	0.69
LV GLS	r-value	0.53	0.5	0.48	0.07	0.15
	p-value	**0.006**	**0.01**	**0.013**	0.68	0.46
LV GCS	r-value	0.54	0.45	0.5	0.01	0.02
	p-value	**0.004**	**0.02**	**0.009**	0.95	0.91
LA Es	r-value	− 0.27	− 0.3	− 0.21	− .39	− 0.17
	p-value	0.18	0.13	0.31	**0.02**	0.4
LA Ee	r-value	− 0.24	− 0.32	− 0.16	− .32	− 0.36
	p-value	0.23	0.1	0.45	0.06	0.07
LA Ea	r-value	− 0.007	− 0.1	− 0.02	− .41	− 0.3
	p-value	0.97	0.78	0.94	**0.02**	0.14

Correlations of tissue characteristics and functional parameters. R-values in bold indicate statistical significance. Es: reservoir strain; Ee: conduit strain; Ea: boosterpump strain; GLS: global longitudinal strain; GCS: global circumferential strain; LGE: late-gadolinium-enhancement; LV: left ventricular; LV myocardial volume; LGE: left ventricular myocardial volume without LGE

In univariate regression analyses, LV matrix volume was univariably associated with the recovery of LV GLS (p = 0.006), LV GCS (p = 0.026), LA Es (p = 0.021) as well as LA Ea (p = 0.008) (Table 4). Similar findings were made for LV cellular volume with the recovery of LV GLS (p = 0.002), LV GCS (p = 0.012) and LA Ea (p = 0.01).

**Table 4:** Regression analysis of CMR-derived myocardial fibrosis over 1 year

Independent variable	Dependent variable	Univariate regression coefficient	p-value
LV matrix volume	Δ GLS	0.42	**0.006**
	Δ GCS	0.44	**0.03**
	Δ LA Es	− 0.44	**0.02**
	Δ LA Ea	− 0.3	**0.008**
LV cellular volume	Δ GLS	0.57	**0.002**
	Δ GCS	0.49	**0.01**
	Δ LA Ea	− 0.51	**0.01**

Regression analyses to assess the influence of CMR-based tissue composition strain alterations. All delta values (Δ) were calculated (baseline–follow up). Es: reservoir strain, Ea: boosterpump strain, GLS: global longitudinal strain, GCS: global circumferential strain, LA: left atrial, LV: left ventricular

**Fig. 6:**
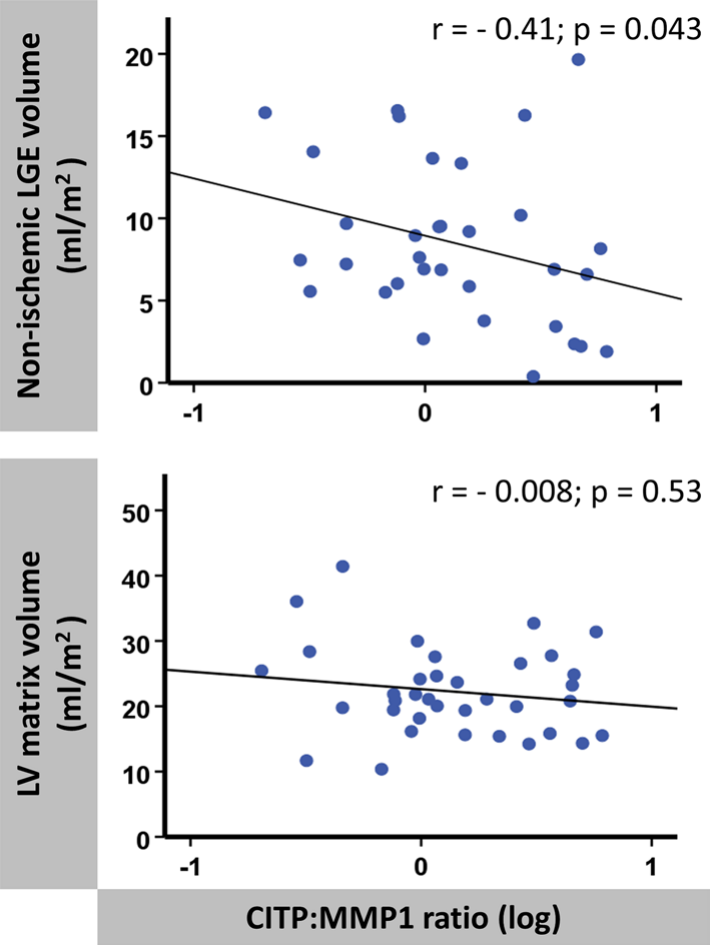
Correlations of CITP:MMP1 ratio with myocardial tissue analyses. Scatter diagrams displaying correlations of non-ischemic late-gadolinium-enhancement (LGE) volume and LV matrix volume both given in ml/m^2^ with the logarithmised (log) ratio of serum carboxy-terminal telopeptide of collagen type I to serum matrix metalloproteinase-1 (CITP:MMP1)

Regarding associations of CMR imaging parameters and biomarkers, all ventricular strain parameters as well as atrial reservoir and boosterpump strain values at baseline correlated significantly with NT-proBNP levels. Likewise, LV matrix volumes, LV cellular volumes and LV myocardial volume LGE- showed associations with NT-proBNP levels (p < 0.01 for all) while for the absolute amount of non-ischemic LGE volume only a statistical trend was documented (p = 0.055). Furthermore, a significant correlation between CITP:MMP1 ratio and non-ischemic LGE volume was observed (r = − 0.41; p = 0.043), whereas there was no statistical association of CITP:MMP1 ratio with LV matrix volume (r =  0.008; p = 0.53) (Fig. 6).

## Discussion

This study aimed at investigating myocardial function and structure in patients with severe AS following TAVR using non-invasive CMR imaging. Several notable findings should be considered: (1) TAVR leads to a regression of both LV matrix and cellular volumes. (2) Myocardial performance both of the ventricles and atria recovers 1 year after intervention. This functional and morphological remission from severe AS is paralleled by improvements of heart failure quantified with clinical examinations and laboratory-testing. (3) Amongst the various changes in morphology CMR-based LV matrix volume extent representing a surrogate marker for diffuse fibrosis correlates with functional myocardial impairment before intervention and predetermines recovery of cardiac performance parameters following TAVR.

TAVR implantation results in an immediate improvement of cardiac loading conditions enabling both functional and structural changes [24, 25]. On a histological level, regression of cellular hypertrophy and interstitial fibrosis content have been described in former biopsy-based studies [26, 27]. In contrast, CMR imaging offers great potential to assess myocardial tissue composition non-invasively, overcoming limitations of myocardial biopsies that carry the risk of the procedure and only allow a small extraction of myocardial tissue [12]. At present, different CMR-based analysis techniques and approaches are available to measure myocardial tissue composition: First, ECV% analyses determine a relation between cellular and extracellular myocardial compartments enabling calculations of LV matrix and cellular volumes. While cellular parts mainly comprise of myocytes, fibroblasts and endothelial or blood cells within microvessels, extracellular components include matrix volume and capillary spaces. Importantly, since myocardial collagen volume accounts for the largest parts of extracellular matrix [22] and because robust associations with biopsy based diffuse myocardial fibrosis have been documented [28, 29], LV matrix volume can be assumed as a surrogate parameter of diffuse myocardial fibrosis [22]. 

Second, LGE analyses visualize considerably increased extracellular volume and potential myocardial fibrosis by extracellular accumulation of gadolinium. Especially non-ischemic LGE has been shown to represent advanced and/or irreversible replacement fibrosis and smaller areas of microscarring in AS patients [12, 30]. However, since LGE analyses require clear demarcations of signal intensity, ECV assessments enable precise identification of subtle diffuse interstitial fibrosis and therefore might be the preferable parameter for this purpose [12, 22]. By excluding areas with clearly definable ischemic LGE or non-ischemic LGE from ECV analyses in our study, we aimed at measuring interstitial processes within remaining viable myocardium separately from potentially more severely damaged myocardial regions and consequently suggest four elementary compartments of importance for myocardial remodeling processes: (1) The LV cellular compartment mainly represented by myocytes (2) the extracellular volume within viable myocardium analyzed by LV matrix volume, (3) non-ischemic LGE patterns comprising focal as well as patchy enhancement and (4) ischemia induced irreversibly damaged myocardial areas (ischemic LGE). 

We documented a significant decrease of both LV matrix and cellular volumes, the latter was proportionally larger, resulting in an increased ECV, an absolute decrease of non-ischemic LGE volume but not as a percentage of LV mass and a significant reduction of LV myocardial volume LGE− 1 year after TAVR. Consequently, TAVR entails regression of LV matrix and cellular volumes and at least in parts non-ischemic LGE, which therefore represent plastic components of myocardial structure. However, within LV myocardial volume LGE-especially LV matrix volume regressed relatively less compared to cellular volumes, suggesting that reverse remodeling of disrupted matrix structure is temporally delayed compared to the cellular volume decrease and rising the presumption of a separated and/ or staggered recovery of interstitial and functional compartments following TAVR [31]. Furthermore, the regression of myocardial fibrosis is complex and there may not be a clear discrimination between potentially reversible and definitely irreversible myocardial fibrosis using non-ischemic LGE imaging. It is interesting to speculate whether non-ischemic LGE analyses might partly capture a continuum between stages that are still reversible but may progress to irreversible myocardial damage in the future. This is underpinned by our data that demonstrate a numeric decline of non-ischemic LGE in % of tLV myocardial volume without statistical significance and a regression of absolute non-ischemic LGE volume 1 year after TAVR, that reaches statistical significance. These results are in contrast to previous findings [31] and suggest that non-ischemic LGE not only includes irreversibly damaged myocardium but also tissue that still reverses after intervention. Furthermore, we observed an inverse relation between non-ischemic LGE volume and CITP:MMP1 ratio (as a marker of cross-linked irreversible collagen formation), which on the one hand supports the notion of a partially irreversible character of non-ischemic LGE and on the other hand might suggest potentially reversible qualities of cross-linked collagen deposition within non-ischemic LGE areas. Whether our results can also partly be explained by less presence of irreversible fibrotic changes as compared to Treibel et al. [31] or whether the analysis technique of a 3 SD based enhancement assessment itself may not only capture irreversible fibrosis but also extensively marks non-ischemic LGE including diffuse but reversible fibrotic areas remains speculative. Importantly, both LGE and ECV analyses have been demonstrated to possess substantial prognostic information in AS patients [32, 33]. Consequently, with prognostically unfavorable myocardial fibrosis in AS patients [34], especially the level of CMR-based LV matrix volume and its alterations after TAVR might serve for future optimized risk stratification and could be used as an important decision-making imaging parameter for new therapeutic strategies targeting the extracellular compartment including antifibrotic treatment for a more tailored and effective therapy [12, 35].

The documented strain improvements in our study primarily depict myocardial functional recovery from AS. Since strain measurements have substantial prognostic qualities [20, 36, 37], a recuperation of strain after TAVR could provide important prognostic information and monitor the success of the procedure [38]. The fact that larger LV matrix volumes at baseline entailed greater strain recovery 1 year after TAVR might seem counterintuitive at first sight, however, at second glance higher degrees of myocardial stress and commencing but still reversible diffuse interstitial fibrotic remodeling processes consequently offer greater recovery potential for myocardial contractility after TAVR and could explain these observations. It is noteworthy, that we documented significant improvement of atrial strain, which has been proven to possess high diagnostic accuracy for diastolic dysfunction [39]. In addition, LA strain measurements have recently been proven to hold important independent prognostic information in patients with diastolic heart failure [40]. Consequently, with proven adverse impact of LV diastolic dysfunction on outcome in TAVR patients [41], CMR-FT based atrial strain analyses could represent important parameters for future optimized TAVR patient management.

Despite a close interplay of myocardial tissue composition and function, different domains of cardiac vulnerability possessing independent prognostic information [42] make separated analysis of myocardial tissue and contractile function highly advisable. Consequently, comprehensive and independent analyses of all compartments might even enable the identification of new high-risk groups or could help to better estimate individual procedural risks. Moreover, the implementation of CMR-based strain measurements for more accurate cardiac performance evaluation beyond sole LVEF assessment and especially atrial strain analyses for precise detection of diastolic dysfunction might further improve AS patient management since recent AS management guidelines mainly focus on (LVEF-based) systolic dysfunction or AS-related symptoms for valve replacement recommendations [43]. Finally, combinations of both functional and structural CMR-derived parameters could create new staging classification systems for AS patients undergoing valve replacement [44].

Therefore, comprehensive CMR-based quantification of myocardial deformation and tissue composition is a complex but promising non-invasive clinical tool providing insights into reverse myocardial remodeling and functional recovery from severe AS and may offer prognostic potential for specified and optimized therapy strategies after TAVR. Easy implementation of this modality in clinical routine makes CMR imaging a key tool for monitoring and subsequent management of patients with AS undergoing TAVR.

## Limitations

Patients were recruited during daily clinical practice without excluding other comorbidities. Therefore, alterations of all parameters might be partly attributable to the combination of AS and other comorbidities (e.g. coronary artery disease). However, ischemic areas were excluded from tissue analyses and had no association with myocardial function in our study. Furthermore, an early CMR follow-up examination to assess immediate effects after relief of AS on myocardial performance was missing in the study protocol and only patients who survived 1-year follow-up examination were included to this study, resulting in an immortal time bias. For an even better understanding of cardiac remodeling after TAVR, future research should address short-term and long-term CMR follow-up examinations.

Importantly, patients with contraindications for CMR and/or being unable to lie in a supine position for the time of CMR scanning were excluded from this study. Nevertheless, findings might be even more pronounced with inclusion of potentially sicker patients. Data on reproducibility of CMR imaging parameters are not provided but have been repeatedly proven to possess excellent intra- and interobserver variability by our imaging core laboratory in recent studies [18, 20, 28].

## Conclusion

Functional and structural reverse myocardial remodeling in patients with AS undergoing TAVR can be comprehensively depicted and quantified with non-invasive CMR imaging analyses. Regression of CMR-based myocardial LV matrix volume is accompanied by an improvement in ventricular as well as atrial strain and is associated with myocardial functional recovery from heart failure following TAVR. Clinical validation and potential prognostic implications of the quantified parameters and observed changes will need to be addressed next to identify therapeutic targets and strategies for an optimized management of these patients.

## Data Availability

Regarding data availability, we confirm that all relevant data are within the paper and all data underlying the findings are fully available without restriction and can be accessed at the University Medical Centre Göttingen by researchers who meet the criteria for access to confidential data.

## Abbreviations

6MWT: 6-minute walk test
AS: Aortic stenosis
bSSFP: Balanced steady state free precession
CITP: MMP1: Ratio of serum carboxy-terminal telopeptide of collagen type I to serum matrix metalloproteinase-1
CMR: Cardiovascular magnetic resonance
Ea: Left atrial booster pump strain
ECV: Extracellular volume fraction
Ee: Left atrial conduit strain
Es: Left atrial reservoir strain
FT: Feature-tracking
GCS: Global circumferential strain
GLS: Global longitudinal strain
LA: Left atrium/left atrial
LGE: Late gadolinium enhancement
LV: Left ventricle/left ventricular
LVEDV: Left ventricular end-diastolic volume
LVEDVI: Left ventricular end-diastolic volume index
LVEF: Left ventricular ejection fraction
LVESV: Left ventricular end-systolic volume
LVESVI: Left ventricular end-systolic volume index
MLHFQ: Minnesota Living with Heart Failure Quality of Life
MOLLI: Modified Look-Locker Inversion recovery
NT-proBNP: N-terminal pro-hormone brain natriuretic peptide
NYHA: New York Heart Association
SAx: Short axis
TAVR: Transcatheter aortic valve replacement
TE: Echo time
tLV: Total left ventricular myocardial volume
TR: Repetition time

## Glossary of CMR derived tissue analyses parameters

Total left ventricular myocardial volume (tLV myocardial volume) in ml/m^2^.: Total LV myocardial volume including all cellular and extracellular components and including LGE areas.
Ischemic late-gadolinium-enhancement (iLGE) volume in ml/m^2^.: Ischemia-induced late-gadolinium enhancement generally extending from the subendocardium with varying transmurality. Quantification is based on a 3-standard deviation (SD) threshold of signal intensity for the detection of enhancement.
Non-ischemic late-gadolinium-enhancement (niLGE) volume in ml/m2.: Non-ischemic LGE patterns comprising focal as well as patchy areas within the myocardium. Quantification is based on a 3-standard deviation (SD) threshold of signal intensity for the detection of enhancement.
Left ventricular myocardial volume without LGE (LV myocardial volume LGE-) in ml/m^2^.: LV myocardial volume without LGE potentially representing viable tissue after excluding ischemic and non-ischemic LGE.
Extracellular volume fraction in % (ECV%).: Volume fraction reflecting myocardial tissue that is not occupied by cells. Both ischemic and non-ischemic late-gadolinium-enhancement areas are excluded from ECV% analyses.
Left ventricular cellular volume in ml/m^2^.: Calculated from the product of LV myocardial volume without LGE (LV myocardial volume LGE-) and (1 – ECV%) representing cellular parts and mainly comprises of myocytes.
Left ventricular matrix volume in ml/m^2^.: Calculated from the product of LV myocardial volume without LGE (LV myocardial volume LGE-) and ECV% representing extracellular components including diffuse myocardial fibrosis.
